# Agent‐Based Modeling in Systems Pharmacology

**DOI:** 10.1002/psp4.12018

**Published:** 2015-11-13

**Authors:** J Cosgrove, J Butler, K Alden, M Read, V Kumar, L Cucurull‐Sanchez, J Timmis, M Coles

**Affiliations:** ^1^ York Computational Immunology LabUniversity of YorkYorkUK; ^2^ Centre for Immunology and InfectionUniversity of YorkYorkUK; ^3^ Department of ElectronicsUniversity of YorkYorkUK; ^4^ Charles Perkins CentreUniversity of SydneySydneyAustralia; ^5^ University of California School of MedicineLA JollaCaliforniaUSA; ^6^ GSK Medicines Research CentreGunnels Wood RoadStevenageUK; ^7^ SimOmicsYorkUK

## Abstract

Modeling and simulation (M&S) techniques provide a platform for knowledge integration and hypothesis testing to gain insights into biological systems that would not be possible *a priori*. Agent‐based modeling (ABM) is an M&S technique that focuses on describing individual components rather than homogenous populations. This tutorial introduces ABM to systems pharmacologists, using relevant case studies to highlight how ABM‐specific strengths have yielded success in the area of preclinical mechanistic modeling.

## AGENT‐BASED MODELS: INTRODUCTION AND APPLICATIONS

The model‐based drug discovery and development paradigm is gaining traction in the pharmaceutical industry. There has been a near constant flow of new terms introduced into the literature^1^ in an attempt to capture this phenomenon: “MBDD,”^2^ “model‐facilitated” or “model‐informed drug development,”^3^ “Quantitative and Systems Pharmacology,”^4^ and “pharmacometrics.” Large pharmaceutical companies have begun to review, quantify, and report the successes derived from the adoption of a model‐based strategy, providing a thorough description of its implementation and impact.^5^, ^6^, ^7^ The US Food and Drug Administration (FDA) recently utilized mechanistic model‐based methodologies to design a postmarketing clinical trial^8^; serving as a clear demonstration of the increasing confidence in and adoption of model‐based techniques in pharmacology.

Many of the methodologies utilized to support the deployment of M&S strategies are recurrent across companies, forming a key component of the “learn and confirm” drug discovery and development paradigm.^9^ Such methodologies typically include, among others, pharmacokinetics and pharmacodynamics modeling (PK/PD), statistical design methods, and signaling network reconstruction methods. Such techniques can be applied to various stages of the drug development process, with the capacity to inform experimental design, “go/no‐go” decisions, preclinical development, and portfolio prioritization.

Wider adoption of M & S in novel therapeutic design requires the development of techniques capable of assessing whether a putative target will yield a desired disease outcome.^10^ Capturing heterogeneous biological systems with phenomena occurring across distinct time and length scales in a single model is a challenging yet frequent requirement in target evaluation and selection.

ABM is used sparingly in a pharmaceutical context. However, it is equipped to address the issue of putative target evaluation, and ultimately, to provide a means of consolidating existing mechanistic understanding into a platform for hypothesis testing and decision support. The use of ABM is increasing within the basic, social, and ecological sciences, providing novel insights into complex systems and engineering challenges.^11^

This tutorial focuses on providing an overview of the ABM methodology within a systems pharmacology context. ABM is an M&S technique characterized by emphasis on understanding how population‐level behaviors emerge from the aggregate interactions of *individuals*, both with the environment and each other.^12^ ABMs are composed of individual entities, known as *agents*; each agent exists in a well‐defined *state*, determined by the agent's attributes and location at a specific point in time. A transition into an alternative state is governed by a predetermined *rule‐set* designed to capture the agent's interactions with other agents or the environment. Using these rules, agents calculate how to respond to features and stimuli within their local environment. The aggregate effects of these individual decisions lead to the emergence of system‐wide patterns and behaviors that are not explicitly programmed or intuitively understood from the rules alone. In designing ABMs, hypotheses can be developed as to how individual components or pathways contribute to tissue‐ or organism‐level effects: hypotheses that may be examined further with experimental or clinical studies.^13^

In the following sections we draw on examples from both the basic sciences and the pharmaceutical development process to illustrate how an ABM approach can provide value in the context of preclinical target evaluation. Specifically, we focus on three key areas where ABMs are advantageous to other M&S techniques: the ability to serve as a platform for knowledge integration (Application 1), to incorporate heterogeneity (Application 2), and to model phenomena occurring across different temporal and spatial scales, often through incorporating other modeling techniques in a process known as *hybridization* (Application 3). The application of these strengths is further highlighted in our case study, which demonstrates how an ABM approach is used to systematically analyze two intervention strategies in the autoimmune disease, experimental autoimmune encephalomyelitis (EAE). As the case study progresses, we identify key design and implementation issues associated with the agent‐based approach, as well as engineering‐inspired techniques and design principles available to tackle these issues. The supplementary information provides links to additional ABM tools (**Table S1**), resources and tutorials (**Table S2**) including hands‐on examples for the interested reader.

### ABM Application 1: a platform for knowledge integration, hypothesis testing and experimental design in studies of acquired immunity

As preclinical R&D programs progress, there is a need to translate knowledge between multiple researchers from various disciplines and, despite increasing knowledge, targetable mechanisms are always to some extent incompletely understood. A number of articles have highlighted the difficulties associated with embedding information in equation‐based techniques and suggest that the conceptual models developed to understand a biological system may not be fully represented in the resultant equations.^14^, ^15^, ^16^, ^17^

Adopting an ABM approach permits the consolidation of existing information into a software platform capable of rapid hypothesis testing and provides a means of identifying key features of a mechanistic target.^16^ An ABM approach is advantageous as a knowledge‐integration platform because: (i) An ABM has the ability to capture phenomena occurring across spatiotemporal scales; (ii) the highly visual output of ABMs affords an effective medium for communication within interdisciplinary teams who may not be *au fait* with advanced mathematical notation^18^; and (iii) an ABM permits the emergence of phenomena from low‐level assumptions (discussed further in “Agent Rules”) offering a unique means of identifying knowledge gaps.^13^ When high‐level properties of an ABM do not emerge as anticipated this provides evidence that the hypothesis on which the model is built is false.

The ability of ABM to provide a platform for knowledge integration, hypothesis testing, and experimental design has been used to study a key mechanistic target in vaccinology: the germinal center.^18^ The germinal center is a transient microenvironment where B cells enter a state of accelerated evolution to form a robust humoral immune response against a single pathogen.^19^ Based on constraints obtained from the literature, Meyer‐Hermann *et al*.^20^ exploited the emergent property of ABMs to test the veracity of different hypotheses regarding B‐cell selection. In that study, the authors compared models developed for each proposed theory and were able to reject those failing to reproduce experimentally observed kinetics.^20^ This ABM was further developed to incorporate the effects of Toll‐like receptor 4 (TLR4) signaling on the germinal center and inform experimental studies.^21^ *In silico* simulation was used to develop novel hypotheses and to identify critical timepoints and conditions to test *in vivo*. This combined experimental and theoretical approach yielded a novel mechanistic insight into the impact of TLR4 signaling on the production of high‐affinity antibodies.

### ABM Application 2: a tool to understand the molecular mechanisms contributing to patient variability

The use of M&S techniques to inform clinical trial design can demonstrably reduce the number of required studies and maximize the probability of success, as evidenced by several successful evaluations reviewed in the literature.^3^ However, patient heterogeneity can arise from genetic, molecular, and tissue levels of organization, thus making the anticipation of patient‐specific responses challenging.

Top‐down data‐driven approaches, typically used to find patterns in existing datasets, provide value in addressing the issue of patient heterogeneity by stratifying patients on the basis of efficacy biomarkers, but often fail to provide mechanistic explanations for the disease‐associated patterns that are discovered. Deriving a mechanistic understanding of how heterogeneity can arise in biological systems on a fundamental level provides a better understanding of why one patient will respond differently to another. Equation‐based models such as ordinary differential equations, which typically provide averaged approximations of interactions occurring in well‐mixed space, can incorporate heterogeneity to some extent through parameter perturbation. However, as system complexity increases, modeling cellular heterogeneity may necessitate the duplication of large portions of model structure for each distinct cell population in situations where parameter adjustment alone is insufficient.

The ability to describe individual molecules, cells, or patients provides a useful means of describing the underlying mechanisms, in addition to stochastic events, which contribute to heterogeneity. The ability of ABM to perform “patient‐specific” *in silico* studies has been described in a hypothetical proof‐of‐principle study, which simulated clinical trials of anti‐cytokine therapy.^22^ In this study, key mechanisms relating to dynamics of the innate immune response in the context of systemic inflammatory response syndrome and multiple organ failure were described using an ABM approach. The resulting simulation of treatment regimes produced outcomes qualitatively similar to those reported in the literature, with none of the simulated therapies showing a statistically significant improvement in mortality rates. This simple model did not stratify patient subsets; however, it did provide insight into the robustness of innate immune system responses to varying therapeutic interventions, and shows how negative results yielded from quantitative systems pharmacology studies can inform key decisions in target evaluation.

ABMs have since been used to stratify patients on the basis of mechanistic understanding of spinal cord and vocal fold pathologies. An ABM approach undertaken by Solovyev *et al*.^23^ combined data regarding blood flow, skin injury, inflammation, and ulcer formation to study the propensity of spinal cord injury (SCI) patients to undergo ulcer formation. The simulation predicted a higher propensity for ulcer formation in SCI patients, thus identifying a high‐risk patient subset.^23^ A similar approach was undertaken to develop personalized treatment strategies in the context of vocal fold injury, where a high degree of patient variability can make it difficult to predict patient‐specific disease progression and treatment responses. Li *et al*.^24^ used an ABM approach to consolidate existing information of inflammatory mediators and used this information to determine optimized treatment strategies.

### ABM Application 3: harnessing spatiotemporal resolution to probe mechanistic targets occurring across distinct space and timescales

While systems pharmacology approaches have been successfully applied to tackle key issues in oncology, for instance, with the use of an integrative systems approach to quantify anticancer drug synergy in imatinib‐resistant chronic myeloid leukemia,^25^ clinical trials in oncology still have the highest failure rate in comparison to other therapeutic areas.^26^ In complex processes such as tumor formation, probing targetable mechanisms can be difficult owing to variability arising on multiple scales; cancerous cells adapt at genetic and molecular scales to survive in ever‐changing environments, altering cellular phenotypes and therefore treatment efficacy, as documented in studies of the hypoxic environment in tumor centers.^27^, ^28^, ^29^ From a systems pharmacology perspective, there is a need to develop techniques capable of capturing mechanisms occurring across different time‐ and length‐scales that affect disease prognosis and patient response to treatment.^30^

Ordinary differential equations, which abstract interactions occurring in physical space through the use of contact frequency terms, are suitable in many cases to model biological systems, and can be transformed into partial differential equations to incorporate space explicitly. However, when the biological system of interest is large and complex, it can be difficult to apply these techniques to describe both spatial variation and capture stochastic processes.^31^

ABMs, owing to their explicit representation of space and ability to capture events occurring across multiple scales, can capture phenomena occurring on distinct spatiotemporal scales, resulting in what are typically referred to as multiscale models.^32^ A common method for developing multiscale models is through the hybridization of an ABM with other techniques to simultaneously represent time and length scales at higher or lower orders of magnitude.

Athale and Deisboeck^33^ developed an ABM to examine the effects of a molecular switch (controlled via epidermal growth factor receptor (EGFR) signaling) on tumor spatial dynamics in the brain. The model predicted that this switch could affect tumor expansion, leading to the development of novel hypotheses on the posttranslational regulation of protein expression. A particularly interesting feature of this model is the explicit modeling of feedback effects from single‐cell decisions on overall tumor‐growth dynamics.

A hybrid agent‐based approach incorporating PK/PD modeling approaches was developed to explore the dynamics of tumor growth, infusion, and penetration of the bioreductive drug tirapazamine (TPZ).^34^ In that study, a PK/PD methodology was applied to model factors such as hydrogen ion production, nutrient distribution, and drug concentration, while an agent‐based methodology was used to model each individual cell over space and time, capturing interactions between cells and with the tumor microenvironment. Combining both types of modeling approach enabled determination that the drug was incapable of reaching the edge of the hypoxic region of the tumor, partially due to consumption of the drug as it diffused into the tumor. This example of hybrid ABM‐PK/PD model highlights the ability of ABM to integrate different modeling techniques, overcoming the respective limitations associated with using either modeling approach in isolation.

## CASE STUDY: PROBING THE EFFICACY OF TWO PUTATIVE TREATMENT STRATEGIES IN AN *IN SILICO* MODEL OF AUTOIMMUNE ENCEPHALOMYELITIS

In complex multifactorial pathologies, such as multiple sclerosis, numerous mechanisms may ameliorate disease. Lead target identification and optimization requires a systematic screening of these mechanisms. In this section we describe how the ABM‐specific strengths described above make this modeling approach suitable for exploring two putative intervention strategies in an experimental animal model of multiple sclerosis. We will then utilize this model as a case study for designing and developing an ABM in later sections of this tutorial.

Multiple sclerosis is a neurological disorder in which myelin, a substance that coats the neurons of the central nervous system (CNS) to ensure efficient propagation of neural signaling, is inappropriately targeted by the immune system. The resultant damage leads to a wide range of symptoms occurring across a spectrum of severity.

Much of the current understanding of multiple sclerosis pathogenesis has been obtained from animal models of the disease such as EAE.^35^ This well‐established experimental model has yielded mechanistic insights into monophasic, relapsing‐remitting, or chronic forms of the disorder. Importantly, a murine EAE model has shown that disease is in part mediated through the action of encephalitogenic CD4Th1 cells.^36^ Furthermore, the killing of encephalitogenic CD4Th1 cells in a coordinated effort from regulatory CD4+ and CD8+ T cells mediates recovery from disease.^37^, ^38^ This led to the proposal that inhibition of T‐cell receptor signaling using a monoclonal antibody against CD3 might target encephalitogenic T cells, thereby either preventing EAE or expediting its recovery. Anti‐CD3 therapy was first used to treat transplant rejection (muromonab) and is currently being investigated for the treatment of other autoimmune conditions.

In addition, the importance of the spleen in recovery from EAE has been highlighted in rats: in contrast to the acute symptoms of control animals, splenectomized rats displayed symptoms of chronic disease following EAE induction.^39^ To consolidate experimental findings gained from animal EAE models and to systematically investigate how splenectomy or anti‐CD3 treatment could affect the progression of EAE, an agent‐based model (ARTIMMUS) was developed.^40^ In the following sections we will use ARTIMMUS to introduce the key components of an ABM, illustrate how to develop an ABM using a principled design approach, and show how such an approach has the potential to inform key decision‐making processes in the drug development process.

### Key components of an ABM

**Figure 1:**
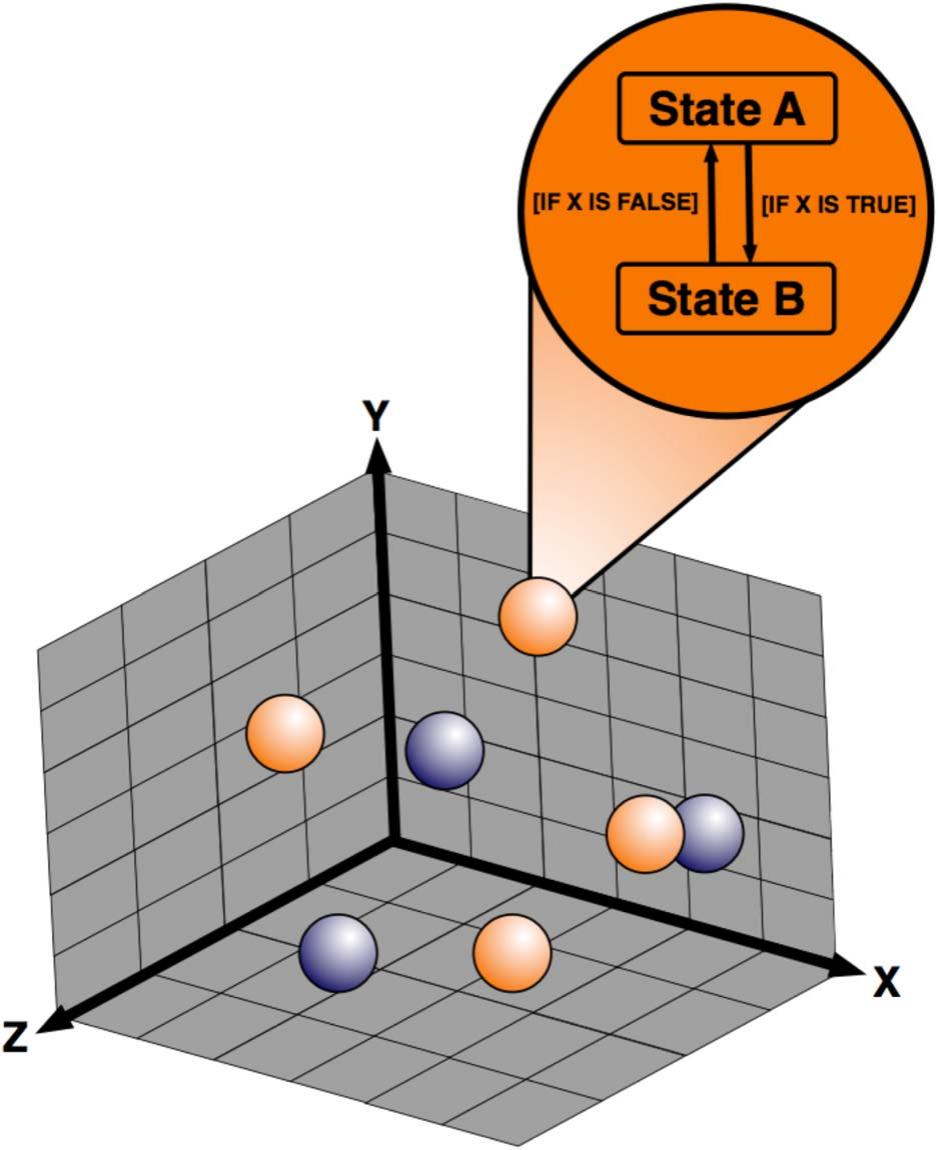
Structure of an ABM: Agents (shown as blue and orange spheres) are individual entities capable of maintaining their associated attributes with respect to their local environment and governing rules. The environment in which an agent exists provides a context for their interactions. The aggregate behaviors of the agents can then lead to the emergence of complex patterns and behaviors.

An ABM contains four main components: 1) agents; 2) the environment within which the agents exist; 3) a rule‐set governing relationships and means of interaction between agents and their environment; and 4) the timescale over which the simulation is executed. The system's overall behavior emerges from the collective interactions of these components over time. These key components and terminology relating to ABM are illustrated in **Figure** 1 and **Table** 1.

**Table 1:** Table of ABM terminology

Entity	An independent element of the model, such as a cell or protein.
Finite state machine	A finite state machine consists of a set of states, which may include substates, some of which are orthogonal (simultaneous states). A finite state machine may exist in only one state for each orthogonal group at a time.
Agent	An autonomous, self‐directed representation of an entity, operating as a finite‐state machine.
Space	Computational representation of the physical spatial compartments within which agents are contained.
Environment	Features in space, which provide a context for agent interactions and behaviors, may contain distributions of molecular concentrations that both influence and can be influenced by agent behavior.
Neighborhood	The local environment in which an agent exists, often described as the agents adjacent to, or in contact with a specific agent.
Neighbor	An agent that exists within the neighborhood of another agent.
Model	A nonexecutable description of a system, which may be described in an abstract manner, or for a platform‐specific implementation as a simulation.
Simulation	An executable implementation of a model specification.
Step	An iteration in time of a discrete‐event simulation.
Hybridization	Using a combination of modeling techniques to capture aspects of the system at different scales in a tractable manner, to overcome the limitations associated with using each technique in isolation.
Multiscale	A model combining processes occurring at different orders of magnitude of time and length.
Richness	The detail contained within an agent, environment or model; comprising internal representations of properties such as cell‐surface protein levels, gene expression, etc.

#### Agents

The agents of an ABM are autonomous, self‐directed entities that are designed to represent individual biological components. Each agent is associated with a set of states in which it may exist, and which define its behavior; this is referred to as a finite‐state machine. Some states are mutually exclusive, while others are orthogonal (simultaneous states), allowing finite state machines to capture complex biological behaviors. Importantly, an agent only has local information available at a given moment in time. Decisions and behaviors therefore arise due to factors in an agent's local neighborhood, or through interactions with neighboring agents.

**Figure 2:**
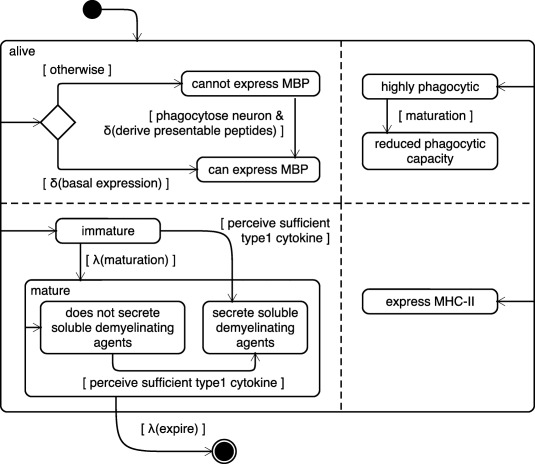
Microglia modeled as agents using the UML. The modeling of microglia in ARTIMMUS. Microglia exist only in the CNS. The only MHC:peptide complex that they present is MHC‐II:MBP. This presentation requires the phagocytosis of a neuron and is probabilistic. A small proportion of microglia expresses MBP immediately, represented by *λ(basal expression)*. This is to reflect the fact that the physiological turnover of neurons (which is not in itself represented in the domain model) will result in their phagocytosis by microglia and the presentation of MHC‐II:MBP complexes. Microglia exist in immature and mature states. While immature they are more phagocytic than when mature. Maturation occurs some time into their lifespan, represented by *λ(maturation),* but may also be induced through perception of a sufficient concentration of type 1 cytokine. Perception of sufficient concentration of type 1 cytokine induces TNF‐α secretion in microglia. Both immature and mature microglia are able to express MHC‐II molecules. Microglia do not exist indefinitely, and expire after some period of time, represented by *λ(expire)*. Figure adopted from Read *et al*.^39^

We now consider modeling a key cell type from our case study, the microglia, as a state machine using the Unified Modeling Language (UML), a set of diagrammatic tools for designing software‐based systems. **Figure** 2 depicts the states in which a microglia may exist. Microglial cells exist in immature and mature states. While immature, they are more phagocytic than when mature. Both immature and mature microglia are able to express MHC‐II molecules. Microglia do not exist indefinitely, and expire after some period of time (depicted as a ringed black circle in **Figure** 2), indicating that the component is destroyed.

#### Agent rules

An agent uses a predefined rule‐set to assess its internal state in response to factors in the agent's local environment or neighborhood. Should an agent be in a situation where the requirement of a rule is met, whether due to a change in the agent's attributes or within a set location in the environment, the state of that agent is changed. An agent's rule set can range from simple Boolean statements operating over the agent's attributes, to more sophisticated mechanisms that relate agent inputs and outputs such as differential equations^16^ and metabolic models.^17^ Agent rules also offer a means of introducing stochasticity through probabilistic events, allowing for an approximation of behaviors in nondeterministic systems.

In our case study we see an example of a rules governing microglia behavior (**Figure** 2). The transition of a microglia from a highly phagocytic state to one of reduced phagocytic capacity depends on the cell undergoing maturation. Maturation occurs some time into their lifespan, represented by *λ(maturation),* but may also be induced through perception of a sufficient concentration of type 1 cytokine. Perception of sufficient concentration of type 1 cytokine induces tumor necrosis factor alpha (TNF‐α) secretion in microglia.

**Figure 3:**
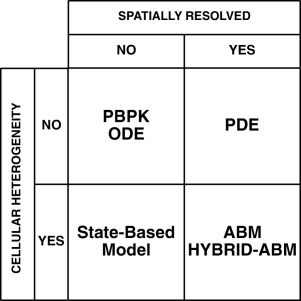
The capacity for various types of model to capture spatial resolution and cellular heterogeneity: When determining the appropriate modeling technique to employ it is important to consider the spatiotemporal scales relevant to the system and the heterogeneity of the entities of interest. Ordinary Differential Equations (ODEs) and Physiologically Based Pharmacokinetic (PBPK) models cannot capture systems with explicit spatial resolution (although compartmentalized systems are possible), relying on the abstract notion of well‐mixed space. Partial Differential Equations (PDEs), and thereby, coupled systems of ODEs, are capable of spatial resolution, but to capture heterogeneous cellular phenotypes is often intractable. State‐based modeling approaches enable heterogeneous phenotypes among cell populations but cannot in themselves capture spatial resolution (although they can model multiple, spatially disconnected compartments). ABMs incorporate state‐based systems in spatial environments; as such, ABMs can capture both heterogeneous cell populations with an explicit notion of space and time.

#### Agent environment

ABMs are usually spatially resolved, with agents occupying specific locations in space rather than existing in a well‐mixed continuum. Space in an ABM is analogous to the physical spatial environments within which biological entities are contained and interact, enabling complex biological structures to be captured within the model. ABMs can contain multiple different spatial environments, typically referred to as compartments, and can allow agents to move between them. In ARTIMMUS a microglia may only reside in the CNS compartment while other agents such as T cells may migrate between different compartments.

#### Timescales and granularity

Time is typically calculated within an ABM by dividing the total time over which the biological system is being simulated into discrete time‐steps. At each time‐step, an agent must determine how to respond to factors in its environment, with respect to its associated rule‐set and current state. In ARTIMMUS each agent must determine how to respond to external stimuli on the basis of its current state and governing rule‐set every 10 minutes. Thus, the agent may show different responses to the rule‐set throughout the course of the simulation. In addition, ABMs can incorporate phenomena occurring at different timescales. Population‐level ordinary or partial differential equations can be used to represent soluble factors or small‐scale molecules, and these can be solved on a per‐agent basis within an ABM.^53^ Modeling these small‐scale factors through population approaches instead of as explicit agents can save considerable computational power. Furthermore, such multiscale modeling reduces the computational expense incurred if the highest resolution timescale were to be used for each phenomenon

**Key Components of ARTIMMUS**

**Agents**

**Figure 4:**
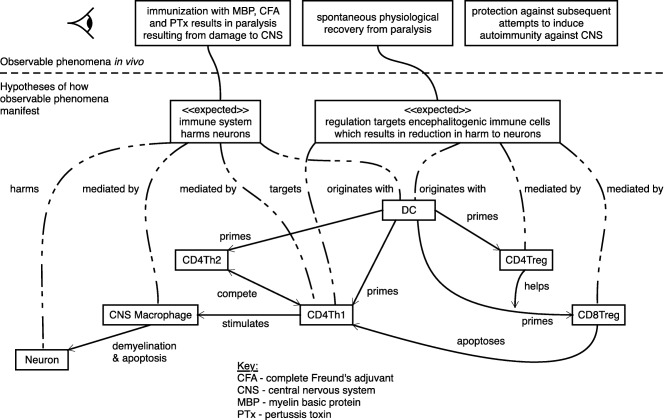
Capturing the emergent phenomena of EAE: An expected behaviors diagram sets the research context of the ABM. This is achieved by depicting the phenomena observed in the murine EAE model, and the behaviors manifesting from cellular interactions hypothesized to be responsible for them. Figure adopted from Read *et al*.^40^

CD4Th1 and CD4Th2 cells, CD4Treg and CD8Treg cells, microglia, dendritic cells, and neurons were identified as having critical roles in the emergence of EAE are included in ARTIMMUS as agents. **Figure** 4 provides an abstract depiction of the major cell types involved in EAE autoimmunity and its associated recovery.

**Agent Rules**

Following *in silico* immunization, agents respond to features in their local environment based on low‐level rules informed by the relevant literature as specified in **SUPPLEMENTARY FIGURE** 1 of Read *et al*.^40^ An example is provided in **Figure** 2 to illustrate this.

**Environment**

**Figure 5:**
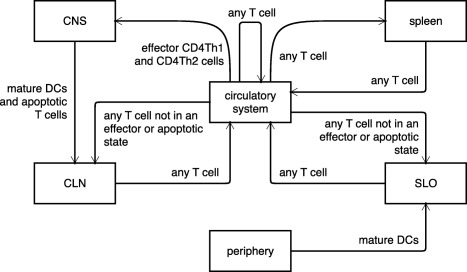
Spatial compartments within ARTIMMUS: The spatial compartments of the domain model, and the manner in which cells may migrate between them. Figure adopted from Read *et al*.^40^

An agent may exist in one of five spatial compartments: the CNS, the draining cervical lymph node (CLN), the spleen, a secondary lymphoid organ (SLO), specifically a peripheral lymph node and a connecting circulatory system. This information is summarized diagrammatically in **Figure** 5.

**Time**

The simulation starts with immunization and typically runs for 60 days postimmunization. Time is discretized into 10‐minute steps.

#### Constructing an ABM

As with any systems pharmacology approach, ABMs are not without their limitations. ABMs are designed to directly capture attributes and states of individual components; population counts and rates of change emerge during simulation execution rather than being explicitly specified. Accordingly, ABMs can be difficult to describe succinctly, and they may require more data to develop, and significant software engineering expertise to implement. For reasons discussed in “Calibrating ABM” and “Exploring Simulation Behavior,” an ABM must be executed multiple times to obtain representative outputs and to ensure robust development and confidence in results.^41^, ^42^ As such, ABM development can require significant time and computational infrastructure. Throughout this section we describe how the key components from the previous section are developed into an ABM by dividing the process into three key stages: design, implementation, and validation. We illustrate how some ABM‐associated issues can be mitigated through the application of well‐established engineering practices, and detail the approaches used in the design of ARTIMMUS.

### Stage 1: designing an ABM

#### Principled design frameworks as standardized protocols for ABM development

The adoption of standardized software practices is a necessary step in ensuring that biomedical software is readable, scalable, and usable.^43^, ^44^ Principled design frameworks that aim to enforce a robust model development methodology facilitate these goals. Grimm *et al*.^45^ proposed a “three‐block” standard protocol for describing ABMs termed ODD (overview, design concepts, and details). The three blocks are subdivided into seven stages: Purpose, State variables and scales, Process overview and scheduling, Design concepts, Initialization, Input, and Submodels. A framework for the development of complex systems models, independent of either domain or modeling techniques, termed the CoSMoS (Complex System Modeling and Simulation) process, has since evolved comprising an iterative process of model refinement and implementation.^46^

Irrespective of the specific protocol, a principled design framework must provide a focus on the underlying research question and current biological understanding. This is particularly relevant when one considers that drug development relies heavily on the quality of published preclinical data^26^; however, studies conducted by Amgen (Thousand Oaks, CA), with a view to confirming published results important to their R&D efforts, could only reproduce similar findings in 11% of cases.^26^ If an ABM is to be used as a means of knowledge integration, then the inclusion, or omission, of specific data used to inform a model should be appropriately justified. An argumentation structure can ensure that the thought processes behind model composition and design decisions are transparent and readily available for scientific scrutiny.^47^ Such an approach takes inspiration from critical systems engineering, e.g., aircraft design, where key decisions are presented as arguments over evidence.^47^

Although there is currently no clear consensus on a standardized protocol for developing ABMs, key features remain consistent across most methodologies: (i) designing an ABM, (ii) implementing an ABM, and (iii) validating an ABM. These processes can be further subdivided, using existing CoSMoS terminology^46^ as follows: (1) defining a research context; (2) developing a nonexecutable model of the underlying biological system: the domain model; (3) developing a simulation specification: the platform model; (4) implementing a simulation: the simulation model; (5) calibration and analysis of the simulation, and (6) simulation validation. In the following sections we further describe these steps and show how they have been applied to the development of ARTIMMUS.

#### Defining a research context

The design and implementation decisions made when constructing an ABM are influenced by the overarching scientific objectives of the work, and simulation results are interpreted in this context. As such an ABM should not be considered a “general purpose” description of a system. By extension, an ABM should not be used outside of its original scope, or within a different research context, without considering whether the existing abstract representation of the system is still fit‐for‐purpose.

It is important to determine whether an ABM approach is the appropriate technique to apply for a given problem. As highlighted under “Constructing an ABM,” above, the technique is not without limitations and therefore the modeling approach that is most suited to the research question should be applied. A basic scheme for selecting a bottom‐up modeling approach is presented in **Figure** 3.

#### Developing a domain model

The initial stage of ABM development should scope the extent of the biological system to be captured. This entails creating a “domain model,” a nonexecutable model focusing purely on biological concerns, disregarding any consideration of how the biology will actually be implemented and simulated. The domain model should describe the states, relationships, and methods of interaction (the rule‐set) for the biological entities being captured. The ARTIMMUS domain model describes the cell types to be captured, how their interactions influence one another's behavior, and the environment in which they exist, but does not specify how they are to be implemented.

#### Developing a platform model

The next stage in constructing an ABM is to delineate how the domain model is to be implemented, an artifact we term the platform model. This is achieved by abstracting biological processes into mathematical constructs to move from the domain model towards a simulation. There are numerous ways in which a domain model might be implemented as a piece of software, the platform model represents the software specification that principled software engineering frameworks advocate creating, as inappropriate decisions and abstractions can render the simulation an inadequate reflection of the biology.

The biological concepts captured in the domain model are translated into a software specification, and the architecture and organization of the simulation software are designed. It is important that any explicit reference to emergent system‐level properties that might be captured in the domain model, used to indicate how these properties emerge from the interactions of the biological components, are omitted from the platform model. Such properties should emerge in the simulation, thereby validating it as a suitable abstraction of the biology. **Figure** 4 depicts how broad system‐level behaviors in ARTIMMUS are hypothesized to emerge from effects that cell populations exert on one another, but was not used in implementing ARTIMMUS; **Figure** 2 was used, depicting the dynamics of an individual microglia.

A further key consideration for a platform model is how users will interact with the simulation. Graphical User Interfaces are useful for assessing whether the code and individual agents perform as expected at any given moment in time, and offer an intuitive understanding of what is happening in the simulation.

**Designing ARTIMMUS**

**Employing a Principled Design Framework**

The CoSMoS framework was employed to develop ARTIMMUS in a principled manner.^46^

**Defining a Research Context**

ARTIMMUS was developed to consolidate the current mechanistic understanding of the murine EAE model and the resultant simulation was designed to systematically probe two intervention strategies: splenectomy and anti‐CD3 treatment.

**Choosing the Correct Technique**

Similar research contexts have been addressed successfully using a differential equation approach by Fousteri *et al*.,^48^ who utilized the type 1 diabetes (T1D) PhysioLab platform, developed to reproduce T1D pathophysiology in the NOD mouse, to make predictions of the impact of timing of the application of nasal insulin to prevent the onset of T1D by simulating features of untreated pathogenesis and disease outcomes for multiple interventions. In the case of ARTIMMUS, however, an ABM approach was warranted due to the heterogeneity of cell populations, the need to account for phenomena occurring on distinct time scales, and features in the cellular environments hypothesized as being important contributors to EAE.

**Developing a Domain Model**

As specified by CoSMoS, the purpose of a domain model is to consolidate current understanding of the biological system with no consideration to how the model might be implemented. Key phenomena and cell types, the dynamics of each cell type, and how their actions integrate to constitute onset and recovery from EAE were explicitly documented in a series of diagrams based on an extended version of the UML.^16^, ^42^ This model was developed with extensive input from experimental collaborators to ensure that the domain model is an adequate representation of the murine EAE model.

**Developing a Platform Model**

With the domain model upon which the model will be based agreed, the next series of UML‐based diagrams concerns their implementation as a simulation (referred to as the platform model in this tutorial). The model details the implementation of the structures, behaviors, and interactions identified in the domain model in a way that naturally translates to simulation platform technologies using the UML. ARTIMMUS employs a 2D lattice‐grid for spatial representation in each compartment and these compartments are networked, allowing cells to leave one compartment and to enter another. Within these lattice grids an agent may move to any of the eight grid spaces surrounding the one in which it currently resides, or it may remain stationary. Between each grid space cytokines and soluble factors represented as concentrations that are subject to decay and diffusion.

### Stage 2: implementing an ABM

#### Simulation Model

The simulation model represents a computer code implementation of the platform model of Stage 1. Code development can be time‐consuming and require specific software engineering expertise; thus, to expedite matters an increasing number of toolkits and ABM‐specific software libraries have become available. These offer programmers a high level of control in development while providing commonly required functionality and data‐structures. A list of commonly used development tools is provided in **Table S1**.

During simulation implementation, state transitions detailed in the specification are typically expressed using control (“if – then”) statements using a programming language. Languages of the Object Oriented programming (OOP) paradigm such as JAVA^49^ have proven a popular and natural means of implementing ABM.^50^ OOPs define *classes*, which are templates for program components and describe how they interact. A class is *instantiated* to obtain an *object* and its associated individual state. Each object is responsible for storing and maintaining its own data, while classes provide functions to strictly control how modifications are made to object data, promoting logical and well‐structured code.

At each discrete time‐step of the simulation (refer to “Timescales and Granularity”) factors in the agent's environment may change, prompting an agent to respond accordingly. Care must be taken to determine the order in which agents are processed, as this could introduce bias. In many instances a randomized schedule is implemented to ensure that one agent's decisions are not prioritized over another's.

#### Calibration

A simulation will contain a number of parameters that control the mathematical constructs used to represent the agents. As these constructs may not translate directly to the biological system, it is often not possible to obtain an exact experimental value for a simulation parameter. Additionally, through the process of abstraction, a parameter controlling one mathematical construct may be accounting for additional factors, which occur in the biological system but have been omitted from the model. For example, ARTIMMUS's “type 1 cytokine” abstracts the function of several cytokines that promote a type‐1, cytotoxic adaptive immune response, such as interferon gamma (IFN‐γ), interleukin 2 (IL‐2), and lymphotoxin alpha (LT‐α). The calibration process identifies simulation parameter values that best align the simulation's behavior with that observed in the experimental system.

Calibration is an important step, as it establishes the baseline behavior of the simulation, to which the results of future *in silico* investigations are compared. Procedures to fit simulation results to biologically derived responses have been described in a study of the life cycle of *Mycoplasma genitalium*.^52^ In brief, the authors fitted their whole cell computational model to datasets spanning metabolomic, transcriptomic, and proteomic information. The resulting parameter values were then validated by determining how well independent experimental datasets were reproduced for multiple cellular functions. However, the effective calibration of complex ABM is an open research area, often requiring the use of sophisticated heuristic optimization technologies.^52^

**Implementing ARTIMMUS**

**Simulation**

The simulation was implemented in the JAVA programming language and compatible MASON simulation framework.^46^ Each cell type identified in Stage 1 was implemented as a Class, based on its state machine diagram (e.g., **Figure** 2).

**Calibration**

ARTIMMUS was calibrated against two experiments^53^: the physiological recovery of mice induced into EAE, and labored recovery following abrogation of CD8Treg capacity to apoptose encephalitogenic CD4Th1 cells.

### Stage 3: validating an ABM

#### Mitigating uncertainty introduced by implementation

Heterogeneity and stochasticity are key benefits of an agent‐based approach, and as with biological systems, repeat experiments can lead to differing results. This variation is termed “aleatory uncertainty” (AU).^54^ Multiple simulation executions must be performed to ensure that the results collected are representative of a simulation experiment, and not simply random variation. Empirical methods exist to establish the relationship between the number of simulation replicates and the effect of stochasticity on aggregated or averaged results,^41^, ^42^, ^55^ but these can be computationally costly to perform. They operate by contrasting samples of simulation responses obtained under the same experimental conditions, thereby estimating the influence of stochastic variation on the distribution of simulation results.

#### Exploring simulation behavior

Prior to using a simulation for *in silico* experimentation, it is important to appreciate how sensitive it is to perturbations in parameters. Such an analysis can be performed through application of sensitivity analysis (SA) techniques.^56^ Through a systematic exploration of the parameter space, simulation inputs that have an influential effect on simulation behavior are identified and quantified, aiding the biological interpretation of simulation results; simulations that critically depend on estimated parameter values should be treated more cautiously.

SA techniques are split into two categories: local and global analysis. Local analysis techniques examine how robust the simulation is to a perturbation of a single parameter value. However, local SA techniques cannot reveal compound effects where one parameter's influence is dependent on the value of another. Such effects may be elucidated using global analysis techniques that perturb multiple parameters simultaneously. For complex models containing a large number of parameters, tractability of the parameter search space is an important consideration. Statistical techniques need to be applied that provide an efficient exploration of the parameter space. Two global analysis parameter sampling techniques, LHC (Latin‐Hypercube) and eFAST (Extended Fourier Amplitude Sampling Test),^41^ have been successfully applied to the analysis of biological ABMs^57^ to determine parameter sensitivity as other parameters are varied.

In addition to highlighting simulation sensitivity to particular parameters, SA techniques can identify key simulated biological pathways.^41^ In the context of preclinical mechanistic modeling, SA techniques can help highlight where a single or combination strategy is required. Marino *et al*.^58^ developed a novel SA approach that explored simulation behaviors, examining how the number of T cells leaving the lymph node affects the bacterial load present in the lung. Uniquely, this approach demonstrates that inputs into SA need not be simulation parameters, but may be emergent properties within the simulation. Separately, SA techniques were also used by Ray *et al*.^59^ to explore a key component of *Mycobacterium tuberculosis* infection: granuloma formation. Granulomas are aggregates of immune cells that can determine host response to infection. Previous experimental work highlighted how cytokine TNF‐α plays a key role in the formation and maintenance of these structures, but the dynamics of this system were difficult to discern using solely experimental techniques. Through the use of ABM, Ray *et al*. isolated and examined five separate functions of TNF‐α, a separation not possible experimentally. They predicted that multiple TNF‐α activities and macrophage activation are key contributors to the control of infection within a granuloma.

#### Experimental validation

While the emergence of expected or observed biological phenomena is one indication that the ABM is an adequate representation of the biological system it captures, it is also important, where possible, that ABM undergo experimental validation.

This is illustrated by Peirce and colleagues^60^ in a model of therapeutic adipose‐derived stromal cell (ASC) trafficking during ischemia. The model design is supported by the simulator's ability to appropriately reproduce important aspects of ischemia and trafficking behavior. Subsequent simulations revealing the necessity for an unknown selectin‐binding molecule to achieve ASC extravasation prompted further *in vitro* experimentation, which confirmed that a subpopulation of ASCs slowly rolled on immobilized P‐selectin further validating the simulations predictions while gaining novel biological insights.

**Validating ARTIMMUS**

**Mitigating Uncertainty**

An aleatory uncertainty analysis^41^ revealed that 500 simulation replicates were needed for each experiment to reduce the influence of aleatory uncertainty.

**Robustness Analyses**

A form of local sensitivity analysis, which reveals how far each individual parameter may be perturbed from calibrated baseline values before scientifically significant deviations in simulation behavior occur, was performed.^53^ The analysis revealed that the majority of calibrated parameters were robust to perturbations.

**Global Sensitivity Analysis**

The importance of various simulation components and pathways was assessed through application of a global sensitivity analysis.^54^ Latin Hypercube Sampling was employed to efficiently sample parameter space, and partial rank correlation coefficients were calculated to determine how parametric variation correlated with changes in simulation behavior.^59^ The analyses lead to a number of insights into the system. For example, the relatively low influence of parameters pertaining to the killing of encephalitogenic CD4Th1 cells by regulatory CD8 T cells suggested a considerable redundancy in this regulatory pathway's ability to ameliorate autoimmune behavior.

### Using ARTIMMUS to inform intervention strategies for EAE

Calibration and sensitivity analysis data suggested that ARTIMMUS constitutes an appropriate representation of murine EAE. Consequently, two interventions were investigated: anti‐CD3 treatment and splenectomy. We summarize them in this section.

#### Splenectomy intervention

To simulate a splenectomy in ARTIMMUS, the entire spleen spatial compartment and its contents (including cells) are removed from the simulation, which prevents cells from entering or leaving it. This facilitated an analysis of the role that the spleen has in EAE onset and recovery. It was shown that the spleen is a major site of T regulatory cells (Treg) priming and that splenectomy significantly reduces CD4+ and CD8+ Treg population sizes, leading to a reduced capacity to completely abrogate encephalitogenic CD4Th1 populations, which can lead to their reexpansion.^40^ Splenectomized groups suffered a higher mortality rate and had a greater tendency towards relapsing clinical disease.

#### Anti‐CD3 therapeutic intervention

As a T‐cell‐mediated autoimmune disease, EAE is potentially treatable using anti‐CD3 antibodies. Such an intervention, with varying efficacies, can be simulated through ARTIMMUS's agent‐based technology. A probabilistic test is performed whenever cognate T cell and antigen binding (in the form of a peptide bound to an MHC molecule) is attempted. In a treatment simulation, an intervention is represented through higher probabilities of preventing this binding taking place. In a control simulation the lack of intervention is represented with an efficacy of 0% (i.e., with zero probability of peptide‐to‐MHC binding prevention).

ARTIMMUS was used to simulate different anti‐CD3 antibody dosing regimens administered at the time of encephalitogenic T‐cell expansion (day 4), or with the onset of clinical symptoms (day 15). *In silico* analyses of putative treatment regimens showed that anti‐CD3 monoclonal antibody (mAb) treatment is not uniformly beneficial in treating EAE. Regardless of administration time, only treatments leading to 80% inhibition of CD3 signaling or greater were effective in reducing the duration of clinical episodes, and preventing clinical relapses. This finding was consistent with clinical trials of anti‐CD3 intervention in new‐onset type 1 diabetes, where large cumulative doses were required for maintaining beta‐cell function^61^, ^62^ and where phase III trials using low doses failed to meet their primary endpoints.^62^

**Figure 6:**
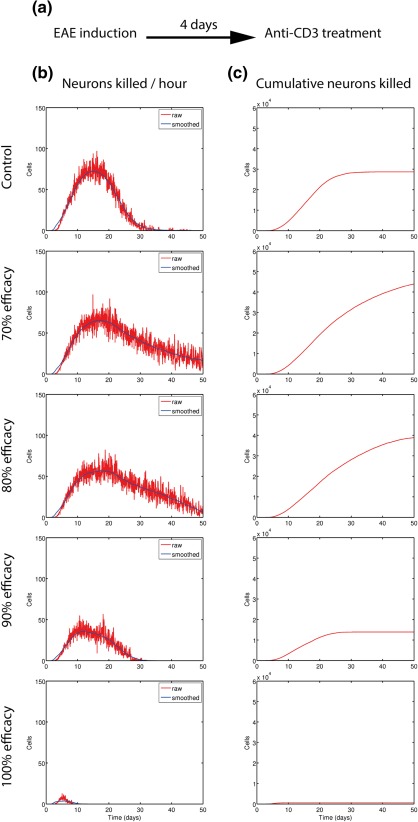
In silico anti‐CD3 treatment result in a lower rate of neuronal death, but a higher number of total neurons killed for some efficacies greater than 80%: (a) EAE was induced at day 0 followed by administration of anti‐CD3 at day 4. (b) The number of neurons killed per hour. (c) Cumulative count of neurons killed for varying efficacies of anti‐CD3 treatment. 100% efficacy blocks all TCR:MHCpeptide bindings, 50% blocks half of all binding events, and 0% represents the control. Figure adopted from Read et al.^41^

Here we show previously unpublished data from the original ARTIMMUS anti‐CD3 intervention experiment, examining how neuronal death relates to varying intervention efficacies. While efficacies of 80% and less can reduce the peak rates of neuronal death, neuronal destruction persists for longer periods of time (**Figure** 6b). The cumulative number of neurons destroyed exceeds control levels for efficacies of 80% and less (**Figure** 6b). These results highlight how interventions that indiscriminately target T cells must be carefully considered, as they can interfere not only with target autoimmune T cells, but the regulatory T‐cell populations that suppress them.

**Computational Resources Required for ARTIMMUS Development and Exploration**

A single execution of ARTIMMUS takes several minutes, depending on the experiment being performed and the machine on which it is executing. Assuming 1 minute per execution, 500 simulation runs would require 8 hours of computational time. The global sensitivity analysis described above used 500 samples of parameter space, and for each sample 500 simulation executions are performed. Hence, this analysis required 173 days of computation time. Given the computational resources required, experiments with ARTIMMUS were conducted on a computational cluster, a network of machines dedicated to performing large‐scale computation. The cluster could process 120 simulation executions in parallel, vastly reducing the time required to carry out experimentation, with most experiments performed within 24 hours.

## DISCUSSION

Agent‐based modeling is a method for constructing computational models focusing on distinct components, their environment, and their interactions, as governed by a set of experimentally derived rules. ABMs can be used to model complex biological systems in which the behavior of heterogeneous individual components, stochastic events, and/or spatiotemporal considerations are important.^23^

As with any quantitative systems pharmacology approach, the ability to integrate knowledge is a key feature of ABMs. The systematic organization of data across scales of interest and different disciplines is nontrivial. The ABM paradigm lends itself well to knowledge integration due to its intrinsically modular organization, ability to capture phenomena occurring on distinct spatiotemporal scales simultaneously, and a highly visual output. The lack of established analytical techniques used in the analyses of equation‐based systems is often perceived as a shortcoming of ABM. However, it is possible to measure and analyze the visual output of ABM in the same manner as wet‐lab systems,^15^ as demonstrated in Butler *et al*.,^63^ with the development of a tool‐chain for enabling existing ABMs to produce emulations of flow cytometry, immunohistochemistry, and gene expression microarrays. An existing simulation of lymphoid organogenesis was modified to produce flow cytometry data, permitting analysis with standard software tools, such that *in silico* and experimental data can be treated and presented in an identical manner. It was demonstrated that such approaches can lead to early indications of model predictions, and intuitively demonstrate these in a biological manner.^63^

Systematically integrating knowledge into a mechanistically coherent output is an important driver for rational experimental design. The capacity to integrate spatial and temporal aspects can facilitate a better understanding of *in vitro* and *in vivo* systems, as demonstrated by Walker *et al*.^64^ That study demonstrated that the calcium‐dependent pattern of wound closure observed for an *in vitro* assay could be quantitatively reproduced *in silico* using simple rule‐based dynamics. Furthermore, differences between *in silico* and *in vitro* models led to predictions for a role in wound‐induced signaling events in urothelial cell cultures, showing the benefits of an iterative modeling and experimental work cycle.

Another key feature of the ABM approach is the ability to capture heterogeneity, such as that of patient populations. The combination of bottom‐up modeling approaches with top‐down data‐driven approaches, such as bioinformatics analyses of high‐throughput genomic data, could present an interesting approach for developing personalized medicine strategies. For example, molecular signatures in the form of biomarker panels could be used as model inputs for subsequent *in silico* simulation of stratified patient populations.^65^

Throughout the tutorial we have discussed the differences between ABM and other modeling techniques. It is important to note, however, that modeling paradigms are not mutually exclusive, and the combination of ABM with other approaches can facilitate the integration of data across different temporal and/or spatial scales. This allows a single model to examine how processes such as molecular diffusion, occurring over a timescale of milliseconds, can affect cellular motility occurring over minutes and hours. ABMs enable phenomena to be captured at the appropriate scale through independent discretization of each scale of interest.

In this tutorial we have highlighted the challenges associated with an agent‐based approach, but reason that such issues can be largely mitigated through the application of well‐established software engineering practices. A number of principled approaches to simulation development and applications are emerging with the aim of ensuring that simulation results are appropriately interpreted.^27^, ^35^, ^37^, ^55^ Achieving a general consensus on a standardized development framework can facilitate model communication, and therefore simulation reproducibility, peer‐review, and repurposing; this therefore should be a fundamental goal in the development of ABM techniques in a systems pharmacology context. CoSMoS, the principled design framework used in the development of ARTIMMUS, for example, shares many key features with the methods for physiological model qualification in drug discovery proposed by *Friedrich and Savic*.^66^ employing concepts from engineering, statistics, complex systems modeling and related fields to address questions of relevance, dealing with uncertainty, dealing with variability, and matching test data in a principled manner.^57^

A key challenge facing the wide acceptance of principled design frameworks is the generation of appropriate model documentation that is clear to an interdisciplinary team, yet concise enough to be practicable.^67^ However, by following existing software engineering principles models can be expressed in discipline‐independent structures and language using visual notations. Visual notations can ensure that the design process is (i) easy for nonspecialists to interpret (provided they are familiar with the syntax), (ii) explicit (i.e., can be interpreted objectively, *not* subjectively), and (iii) accessible. These notations can also be used to provide an argument that the ABM and resultant simulator are a fit‐for‐purpose representation of the underlying biological domain of interest.^69^ Aside from formal mathematical notations,^68^ a number of well‐established notations exist and are addressed in **Table S1**.

The case study in modeling EAE has highlighted how principled simulation development techniques can give rise to well‐engineered simulations, and establishes the link between simulation and the original biological system. We have demonstrated the range of interventions that can be simulated through ABM, from removal of an entire spatial compartment to fine‐grained manipulations of cell‐signaling pathways. Furthermore, the effects of interventions across a heterogeneous and stochastic biological system can be probed: the EAE simulation was used to examine how the range of disease progressions experienced varied with intervention efficacy, be it relatively mild or terminal.^40^

The agent‐based approach, when applied appropriately and in a principled manner, offers unique advantages in the study of highly complex biological systems. Taken together, the studies referred to in this tutorial highlight how ABM has been used to gain novel insights and give an indication into how the approach will be useful for future studies in systems pharmacology.

## Conflict of Interest

J.T. and M.C. are Directors of SimOmics Ltd. L.C.‐S. is currently a permanent employee of GSK.

## Author Contributions

M.C., J.C., J.B., K.A. M.R., L.C.‐S., and J.T. wrote the article; M.C., K.A., M.R., V.K., and J.T. designed the research; J.C., J.B., K.A., and M.R. performed the research; M.C., J.C., J.B., K.A., M.R., V.K., and J.T. analyzed the data.

## Supporting information

Supporting Information

Table S1

Table S2
