# Supplementary material for: Agent‐Based Modeling in Systems Pharmacology
Source: CPT Pharmacometrics Syst Pharmacol. 2015 Nov 13;4(11):615–29. doi: 10.1002/psp4.12018 (PMC4716580; doi:10.1002/psp4.12018)
Supplement: Supplementary file 1 — Supporting Information [file PSP4-4-615-s001.pdf]

```
package sim2d.cell.impl;

import java.util.Set;

import org.w3c.dom.Document;
import org.w3c.dom.Element;

import sim.engine.SimState;
import sim2d.TregSimulation;
import sim2d.cell.Cell;
import sim2d.cell.molecule.MHC_II_MBP;
import sim2d.compartment.Compartment;
import sim2d.molecule.MBP;
import sim2d.molecule.Molecule;
import sim2d.molecule.SDA;
import sim2d.molecule.Type1;

/**
 * Biologically speaking, MHC-II presentation is inducible on Macrophages, but is not presented as standard. However, MHC-II is not explicitly represented here; for our purposes it is
 * redundant, because it's expression dynamics are entirely linked with the dynamics of MBP phagocytosis and expression.
 *
 * CNS Macrophages do not get licensed. Licensing is predominanlty do to with presentation to CD8+ T cells, and there are not MBP-reactive CD8+ T cells in this
 * simulation. Activation of a CNS macrophage (by perception of type 1 cytokines (inf-g)) results in ability to express co-stims and mhc-ii.
 *
 * @author mark
 *
 */
public class CNSMacrophage extends APC_Impl implements MHC_II_MBP
{
	/*
	 * Properties of all CNS Macrophages
	 */
	private static double phagocytosisProbabilityImmature;			// the probability that an APC will phagocytose an apoptotic cell if the APC is immature.
	private static double phagocytosisProbabilityMature;			// the probability that an APC will phagocytose an apoptotic cell if the APC is mature.


	private static double type1RequiredForActivation;				// the quanitity of type 1 cytokines that must be perceived for (an MHC-expressing) APC to express costim molecules.

	private static double sdaSecretedPerHourWhenStimulated;			// the quantity of SDA molecules secreted every hour when this CNSMacrophage is stimulated.
	private static double sdaSecretedPerTimeslice;					// the quantity of SDA molecules secreted every timestep when this CNSM is stimulated.

	private static double basalMBPExpressionProbability;			// the proportion (range 0 - 1.0 ) of CNSM's that, at start of simulation, express MHC-II-MBP.

	/*
	 * properties of CNS Macrophage instances
	 */
	private boolean canExpressMBP = false;
	private boolean canExpressCoStim = false;
	private boolean stimulated = false;
	// this is set to true when the CNS M becomes stimualted through recepit of type 1 cytokines.


	/**
	 * Constructor creates a completely naive CNSMacrophage.  A certain proportion of all new CNSMacrophages are started in a state that presents MBP, this is to reflect the fact that
	 * there will always be some MBP presentation in the CNS. It is also a requirement for the start of EAE, because without local activation the T cells will never perform effector function
	 * in the CNS.
	 */
	public CNSMacrophage(Compartment location)
	{
		super(location);

		if(TregSimulation.sim.random.nextDouble() <= basalMBPExpressionProbability)
		{
			// set up such that CNSM is able to present to Th1 and Th2 cells
			this.canExpressMBP = true;
```

```
				this.canExpressCoStim = true;
				this.setTimeToDeath();

				// set up such that CNSM is not activated and secreting SDA
				this.stimulated = false;				// this is default, but repeated for clarity.
			}
		}

		/**
		 * Constructor should be used to create the initial population of CNSMs in the simulation, and should not be used during the
simulation's run. It sets up the initial population such that it appears that
		 * the simulation has been running for a while, rather than just being run. This involves setting timer values of cells to random
settings within their normal range, rather than starting them all off from
		 * the same point, which will create periodic artefacts in the simulation's run.
		 */
		public static CNSMacrophage createInitialCNSMacrophage(Compartment location)
		{
			CNSMacrophage cnsm = new CNSMacrophage(location);						// create a
normal CNSM.

			// pick a time of death that lies somewhere in the range of now and what would have been chosen in a normal
simulation run.
			cnsm.timeOfDeath = calculateAbsoluteTimeOfDeath() * TregSimulation.sim.random.nextDouble();

			return cnsm;
		}

		/**
		 * Implements any functionality required when a DC goes from an immature state to either tolerogenic or immunogenic.
		 */
		protected void becomeNonImmature()
		{
			setTimeToDeath();												// the
cell will expire some time after it migrates.
		}

		/**
		 * Method handles the secretion of cytokines by this cell into the compartment that this cell occupies.
		 */
		protected void secreteCytokines()
		{
			if(stimulated)				// if the CNS M has been stimulated (through receipt of type 1 cytokines) then it will
secrete SDA.
			{
				compartment.receiveSecretedMolecules(SDA.instance, sdaSecretedPerTimeslice, this);
			}
		}

		/**
		 * Returns true when the APC is both capable of expressing MHC-II molecules and contains the MBP peptide.
		 */
		public boolean getExpressing_MHC_II_MBP()
		{		// note that we are assuming here that MHC-II expression is constitutive.
			return canExpressMBP;
		}

		public boolean getExpressing_CoStimulatory()
		{		return canExpressCoStim;		}


		/**
		 * Returns true if this APC is expressing any MHC molecules.
		 */
		public boolean expressingMHC()
		{
			return getExpressing_MHC_II_MBP();										// this
is the only form of MHC-peptide expressed by CNS macrophages.
		}



		/**
		 * This method handles the perception of cytokines within the compartment.
		 *
		 * there are the following options (taking costim expression as an example)
```

```
	 *
	 * expressing costim? || awaiting expression of costim?
	 * no  || no   => set time to express
	 * no  || yes  => do nothing (definitely do not set back the time to expression!)
	 * yes || no   => relicensing, set time to unexpression further back (reset it)
	 * yes || yes  => this should not be possible, time to expression is set to inf. when it passes.
	 */
	protected void perceiveMolecules(TregSimulation sim)
	{
		double quantity = compartment.getConcentrationMolecule(Type1.instance, this);
		if(quantity >= type1RequiredForActivation) // if there are enough type1 cytokines, and if the APC is expressing 
MHC, then become licensed for costims.
		{
			/*
			 * This is done even if the cell is not expressing MHC to prevent a never starting loop. CNS Macrophages 
will only express mhc if they phagocytose
			 * a CNS cell. CNS cells only die when there is SDA around, and if the only cells that secrete SDA are 
CNS macrophages then the loop never starts.
			 */
			stimulated = true;
		// this means the CNS Macrophage will start to secrete SDA
			becomeNonImmature();
		// this call only sets a time to death, but we call it here for consistency. It does not upregulate co-stims.
			// receipt of type 1 cytokines allows cell to express co-stim molecules.
			if(canExpressCoStim == false && expressingMHC() == true)
			{
				canExpressCoStim = true;
			}
		}
	}


	/**
	 *  Called when an APC is to phagocytose another (given) cell. This method is not responsible for removing the cell from the 
compartment, that is
	 *  handled by cell.driveMHCPresentableMolecules, which also returns the molecules that are MHC presentable from the 
apoptotic cell.
	 */
	public void phagocytoseCell(TregSimulation sim, Cell cell)
	{
		if( cell.isApoptotic() == false || cell.isDead())				// do nothing if the cell is not apoptotic, or if it has 
already been phagocytosed (but not yet removed from the simulation)
			return;

		if( isApoptotic)
			return;
			// dead APCs can't phagocytose anything.

		/* APCs that are stimulated (and express MHC) are less likely to phagocytose other cells. */
		double probOfPhagocytosis = phagocytosisProbabilityImmature;
		if(expressingMHC())										// if 
DC is expressing MHC molecules (ie, it is stimulated)
			probOfPhagocytosis = phagocytosisProbabilityMature;

		if(sim.random.nextDouble() >= probOfPhagocytosis)	// if we are unstimulated (no MHC) then we will continue, if 
we are stimulated then there is a high chance that we will not phagocytose this cell.
			return;

		Set<Molecule> presentable = cell.bePhagocytosised(sim);
		if(presentable == null)
			return;
		// the phagocytosed cell contained no presentable peptides.

		/* anything UPTO 'probabilityPhagocytosisToPeptide' will result in peptides being derived. Else, save computation 
time and return now instead. */
		if(sim.random.nextDouble() >= probabilityPhagocytosisToPeptide)
			return;

		performPhagocytosisOfCell(presentable);
	}

	/**
	 * Handles the actual phagocytosis of a cell. It is protected, so it cannot be an entry point for the phagocytosis behaviour; there 
are guards that need to be checked first, and these
```

```
	 * are handled in 'phagocytoseCell'. Here the molecules in 'presentable' are examined and CNSMacrophage specific behaviours are created in this method.
	 */
	protected void performPhagocytosisOfCell(Set<Molecule> presentable)
	{
		if(presentable.contains(MBP.instance))				// check if the cell contains MBP.
		{
			if(canExpressMBP == false)				// if molecule not already being expressed.
			{
				canExpressMBP = true;
				setTimeToDeath();
			}
		}
	}

	/**
	 * Method handles events and behaviours required to put the cell into an apoptotic state.
	 */
	protected void becomeApoptotic()
	{
		timeOfDeath = Double.MAX_VALUE;
	// reset clock to infinity.
		/* stop this cell from interacting with others */
		isApoptotic = true;

		// replace this cell with another immature one.
		new CNSMacrophage(this.compartment);				// homeostatic replacement of dead cells with immature ones.

		TregSimulation.sim.removeFromSimulationSchedule(this);		// critical, remove this cell from the simulation's schedule.
		compartment.removeCellFollowingDeath(this);				// remove yourself from the compartment.
	}


	/**
	 * T Cells handle interactions with APCs, so they donot appear here.
	 */
	protected void interactWithOtherCell(TregSimulation sim, Cell otherCell)
	{
		if(otherCell instanceof APC_Impl && otherCell.isApoptotic())		// only deal with APCs here, T cells instigate this though their own step functions.
			phagocytoseCell(sim, otherCell);
	}

	public boolean isImmature()
	{
		return (expressingMHC() == false) && (getExpressing_CoStimulatory() == false) && (isApoptotic() == false);
	}

	public boolean isTolerogenic()
	{
		return expressingMHC() && (getExpressing_CoStimulatory() == false);
	}

	public boolean isImmunogenic()
	{
		return getExpressing_CoStimulatory();
	}

	public boolean isMobile()
	{
		if(isImmature() && (stimulated == false))
			return false;

		return true;
			// all other states result in CNSM mobility.
	}


	/**
	 * Returns true when this cell is apoptotic.
	 */
```

```
	public boolean isApoptotic()
	{
		return isApoptotic;
			// Dendritic Cells do not become Apoptotic.
	}

	/**
	 * Returns true when this cell has been phagocytosed, to prevent it being phagocytosed again in the same timestep. However, since these cells are immediately removed from
	 * the simulation upon becoming apoptotic, this method is redundant here, and hence always returns false;
	 */
	public boolean isDead()
	{		return false;		}

	/**
	 * When this cell leaves an immature state, and starts to express MHC molecules (and potentially co-stims too) then its time of death must be set.
	 * Non-immature APCs do not live forever.
	 */
	private void setTimeToDeath()
	{
		if(timeOfDeath != Double.MAX_VALUE)
	// time of death is already set.
			return;

		timeOfDeath = calculateAbsoluteTimeOfDeath();
	}

	/**
	 * Java bean getters and setters.
	 */
	public double getTimeOfDeath()
	{		return timeOfDeath;				}

	public boolean getExpressingMBP()
	{		return canExpressMBP;		}

	public boolean isExpressing_MHCPeptide()
	{
		return (canExpressMBP);
	}

	protected double getPhagocytosisProbabilityImmature()
	{		return phagocytosisProbabilityImmature;			}

	protected double getPhagocytosisProbabilityMature()
	{		return phagocytosisProbabilityMature;			}


   /**
    * Given the parameters.xml file (represented as a 'Document') this method loads the relevant default values for this class.
    * @param params
    */
	public static void loadParameters(Document params)
	{
		Element pE = (Element) params.getElementsByTagName("CNSMacrophage").item(0);

		phagocytosisProbabilityImmature = 
Double.parseDouble(pE.getElementsByTagName("phagocytosisProbabilityImmature").item(0).getTextContent());
		phagocytosisProbabilityMature = 
Double.parseDouble(pE.getElementsByTagName("phagocytosisProbabilityMature").item(0).getTextContent());

		basalMBPExpressionProbability = 
Double.parseDouble(pE.getElementsByTagName("basalMBPExpressionProbability").item(0).getTextContent());

		type1RequiredForActivation = 
Double.parseDouble(pE.getElementsByTagName("type1RequiredForActivation").item(0).getTextContent());

		sdaSecretedPerHourWhenStimulated = 
Double.parseDouble(pE.getElementsByTagName("sdaSecretedPerHourWhenStimulated").item(0).getTextContent());
		sdaSecretedPerTimeslice = sdaSecretedPerHourWhenStimulated * TregSimulation.sim.timeSlice;
	}
}
```
