# Supplementary material for: Agent‐Based Modeling in Systems Pharmacology
Source: CPT Pharmacometrics Syst Pharmacol. 2015 Nov 13;4(11):615–29. doi: 10.1002/psp4.12018 (PMC4716580; doi:10.1002/psp4.12018)
Supplement: Supplementary file 2 — Table S1 [file PSP4-4-615-s002.docx]

| **Tool** | **Description** | **Associated Link** | **Reference** |
| --- | --- | --- | --- |
| **Principled Design Frameworks** |  |  |  |
| ODD | Protocol for developing agent based models | http://www.ufz.de/index.php?de=10466 | Grimm V, Berger U, Bastiansen F, Eliassen S, Ginot V, Giske J, et al. A standard protocol for describing individual-based and agent-based models. Ecol Model. 2006 Sep 15;198(1–2):115–26 |
| CoSMoS | Protocol for developing complex systems models | http://cosmos-research.org/ | Alden K, Timmis J, Andrews PS, Veiga-Fernandes H, Coles MC. Pairing experimentation and computational modeling to understand the role of tissue inducer cells in the development of lymphoid organs. Inflammation. 2012; 3:172. |
| **Visual Notations** |  |  |  |
| Unified Modeling Language (UML) | A standardised graphical language widely used for specifying agent states and governing rules | http://www.uml.org/ | Bersini H, Klatzmann D, Six A, Thomas-Vaslin V. State-Transition Diagrams for Biologists. PLoS ONE. 2012 Jul 23;7(7):e41165. |
| UML (adapted for biology) | A version of the UML adapted for modeling biological systems |  | Read M, Andrews PS, Timmis J, Kumar V. Modelling biological behaviours with the unified modelling language: an immunological case study and critique. J R Soc Interface. 2014 Oct 6;11(99):20140704. |
| Goal Structuring Notation | A visual notation used to document an argument and its relation to available evidence | http://www.goalstructuringnotation.info/ | Alden K, Andrews PS, Polack FAC, Veiga-Fernandes H, Coles MC, Timmis J. Using argument notation to engineer biological simulations with increased confidence. J R Soc Interface. 2015 Mar 6;12(104):20141059. |
| **Agent Based Platforms** |  |  |  |
| MASON | An ABM-specific software library in JAVA | http://cs.gmu.edu/~eclab/projects/mason/ | Luke S, Cioffi-Revilla C, Panait L, Sullivan K, Balan G. MASON: A Multiagent Simulation Environment. SIMULATION. 2005 Jul 1;81(7):517–27. |
| Swarm | An ABM specific software library in objective C | http://savannah.nongnu.org/projects/swarm | Minar N, Y RB, Z CL. The Swarm Simulation System: A Toolkit for Building Multi-Agent Simulations. 1996. |
| REPAST | An agent-based modeling environment but can be used as a stand alone library | http://repast.sourceforge.net/ | North MJ, Collier NT, Ozik J, Tatara ER, Macal CM, Bragen M, et al. Complex adaptive systems modeling with Repast Simphony. Complex Adapt Syst Model. 2013 Dec 1;1(1):1–26. |
| NETLOGO | An agent-based modelling environment | https://ccl.northwestern.edu/netlogo/ | Wilensky U, Rand W. Introduction to Agent-Based Modeling: Modeling Natural, Social, and Engineered Complex Systems with NetLogo. MIT Press; 2015. 505 p. |
| **Simulation Analysis** |  |  |  |
| SPARTAN | A collection of statistical techniques implemented in R | https://www.york.ac.uk/computational-immunology/software/spartan/ | Alden K, Read M, Timmis J, Andrews PS, Veiga-Fernandes H, Coles M. Spartan: a comprehensive tool for understanding uncertainty in simulations of biological systems. PLoS Comput Biol. 2013;9(2):e1002916 |
| **Model Argumentation** |  |  |  |
| Artoo | A tool used for argumentation, implementing goal structuring notation | https://www.york.ac.uk/computational-immunology/software/artoo/ | Alden K, Andrews PS, Polack FAC, Veiga-Fernandes H, Coles MC, Timmis J. Using argument notation to engineer biological simulations with increased confidence. J R Soc Interface. 2015 Mar 6;12(104):20141059. |
