# Supplementary material for: Agent‐Based Modeling in Systems Pharmacology
Source: CPT Pharmacometrics Syst Pharmacol. 2015 Nov 13;4(11):615–29. doi: 10.1002/psp4.12018 (PMC4716580; doi:10.1002/psp4.12018)
Supplement: Supplementary file 3 — Table S2 [file PSP4-4-615-s003.docx]

| **Platform** | **Description** | **Link to Tutorial** | **Difficulty** |
| --- | --- | --- | --- |
| NetLogo | The NetLogo user manual provides basic information, samples models and tutorials to get users started with agent based modelling | http://ccl.northwestern.edu/netlogo/docs/ | Beginner |
|  |  |  |  |
| REPAST | The REPAST tutorial repository provides extensive documentation on model development and links to third party tutorials | http://repast.sourceforge.net/repast_3/tutorials.html | Intermediate |
|  |  |  |  |
| MASON | The MASON user manual provides a hands on example of how to build a simple social network of a schoolyard | http://cs.gmu.edu/~eclab/projects/mason/manual.pdf | Advanced |
|  |  |  |  |
| All Platforms | A template model which can run on several platforms | http://condor.depaul.edu/slytinen/abm/StupidModel/ | All |
|  |  |  |  |
|  |  |  |  |
|  |  |  |  |
|  |  |  |  |
